# Methods to detect anxiety in Parkinsons’ disease

**DOI:** 10.1007/s00702-025-03068-x

**Published:** 2025-12-04

**Authors:** Anette Schrag, Ruby Lyall, Saghi Arabi, Gabrielle Sheehan

**Affiliations:** Department of Clinical and Movement Neurosciences, University College London, U3, Rowland Hill Street, London, NW3 2PF UK

**Keywords:** Parkinson's disease, Anxiety, Parkinson's Anxiety Scale, Cut-off, validation, screening

## Abstract

Anxiety is a common comorbidity of Parkinson’s disease (PD), but its classification varies widely, partly due to differences in assessment tools. Participants in a study on anxiety mechanisms in PD were assessed using multiple anxiety instruments. For each, Youden indices were calculated, and prevalence rates and agreement with the Parkinson’s Anxiety Scale (PAS) cutoff were examined to establish comparable cut-offs. Associations with health-related quality of life (PDQ-39 scores) were used to assess clinical relevance. 85% of 122 participants reported anxiety in response to a single question, but only 19.7% reported a prior anxiety diagnosis. Using a PAS threshold of 14, 50% were classified as having anxiety. Similar rates were found for the GAD7 (54%) with a cut-off of 4/5 and the MDS-NMS anxiety subdomain (55%) with a cut-off of 13/14, both showing good agreement with the PAS. Lower anxiety rates (22%) were observed with the MDS-UPDRS anxiety item (cut-off 2/3), which had fair agreement with the PAS. Self-reported anxiety and previous diagnoses showed slight agreement with the PAS cut-off. Anxiety classifications using standardised scales correlated with worse PDQ-39 scores, unlike self-reported anxiety. Standardised scales with established cut-offs are preferable to self-report or prior Accepted manuscript diagnoses for identifying anxiety in PD. Aligning cut-offs across different scales may enhance comparability between studies.

## Introduction

Anxiety is a common comorbidity of Parkinson’s disease (PD) (Broen et al. 2016) and has been ranked amongst the key priorities for research by patients with PD. However, it has received relatively little attention in clinical research and practice. It is often not reported by patients or recognised by clinicians due to a variety of causes, including stigma, attribution to physical symptoms or circumstances or misattribution of symptoms (Blundell et al. 2023). Substantial differences in the methods to detect the presence of anxiety in PD exist, and this is at least in part due to differences in use of assessments tools. This is a challenge for research relating to mechanisms anxiety in PD, as full assessment using clinical diagnostic criteria is typical difficult in such settings and identification of anxiety depends on screening tools. Furthermore, it is unclear if methods used in clinical settings, such as a single question about presence of anxiety, a previous diagnosis of anxiety or an item on a composite instrument for assessment of PD symptoms in clinical settings are equivalent and clinically meaningful methods to elicit this important feature of PD. In order to improve detection and comparability of methods to identify and classify anxiety in PD, we therefore compared the results of several methods to identify anxiety and its impact in PD.

## Methods

### Participants

122 participants were recruited from hospital clinics, patient organisations and through snowballing from January 2021 to January 2023. Inclusion criteria were broad, including people with a diagnosis of PD aged 18–89 years. We excluded those unable to give informed consent (e.g. advanced dementia), and any other disorder which would make completion of tasks unfeasible. All participants gave their informed consent to participate to the study, which was approved by the UCL Research Ethics Committee, in accordance with the ethical standards laid down in the 1964 Declaration of Helsinki.

### Assessments

As part of a larger study (https://www.and-pd.com/), we collected demographic information and participants underwent an interview about anxiety symptoms using DSM V criteria. They were assessed using the MDS-UPDRS (The International Parkinson and Movement Disorder Society - Unified Parkinson’s Disease Rating Scale) (Goetz et al. 2008) (adapted for remote assessment by excluding the items ‘rigidity’ (item 3.3) and ‘postural stability’(item 3.12) as the study began during the COVID pandemic), and the MDS-NMS (The International Parkinson and Movement Disorder Society – Non-Motor Rating Scale) (Chaudhuri et al. 2020), and were asked to completed a number of questionnaires within the same week, including the PAS (Leentjens et al. 2014), a 12-item scale that assesses anxiety in patients with Parkinson’s disease, GAD-7 (Spitzer et al. 2006), a 7-item self-rated scale that was developed to screen for generalized anxiety disorder (GAD) in primary care settings, PDQ-39 (Peto et al. 1995), a health status questionnaire that assesses how often people with Parkinson’s experience difficulties in eight dimensions of daily life and EQ-5D-5L (Herdman et al. 2011), health-related quality of life instrument that measures health across five dimensions. Other questions on anxiety included a single question whether they suffer from anxiety, self-report of previous diagnosis of anxiety and any medication used for anxiety.

### Analysis

In order to confirm validity and optimal cut-offs on each scale we conducted Receiver Operating Characteristic (ROC) Analysis and calculated Youden indices (Sensitivity + Specificity – 1; Youden 1950) for each scale point against DSM V Anxiety symptoms. For the PAS the Youden index confirmed an optimal cut-off for the PAS of 14, similar to its validation study (Leentjens et al. 2014 ). For the GAD7 the Youden index was similar for a cutoff on the GAD7 of 5/6 and of 4/5 which is equivalent to the published cutoff for mild anxiety (Spitzer et al. 2006). For the MDS-NMS anxiety subdomain no previous cutoff has been published but Youden indices were similar for cutoffs of 9/10, 13/14 and 21/22. For the MDS-UPDRS the highest Youden index was at 2/3, which indicates anxiety symptoms with some interference with daily life.

Anxiety frequency rates, sensitivity and specificity are presented for the cutoffs on the scales. Agreement with the PAS, as the only validated PD disease-specific scale for anxiety in people with PD, to assess anxiety in patients with PD, was calculated using Cohen’s kappa, with 0.01–0.20 representing none to slight agreement, 0.21–0.40 fair, 0.41– 0.60 moderate, 0.61–0.80 substantial and 0.81–1.00 almost perfect agreement (Cohen 1960).

In order to assess whether the methods identified clinically meaningful differences, health-related quality of life scores were compared between the two groups identified by each method, using the Parkinson’s Disease Questionnaire-39 (PDQ-39) and the visual analogue scale of overall health on the EQ-5D-5 L and the Mann-Whitney test for independent samples using a significance level of 5%.

Data were analysed using the statistical analysis software IBM SPSS 29.0.

### Data sharing

Data available upon request.

## Results

Table 1 shows the characteristics of the participants who had a range of disease duration and severity scores. Whilst 85% reported having anxiety in response to a single question, only 19.7% reported a previous diagnosis of Anxiety. 40.3% had received medication for anxiety or depression, and 37% had a family history of anxiety.

**Table 1:** Demographic and baseline characteristics

Variables	*N* (%)	Mean (SD or range)
Age, years	119	65.1 ± 9.4
Parkinson’s disease duration, years	109	5.5 (0.3 to 18.3)
Sex	119	
Men	69 (58.0)	
Women	50(42.0)	
Anxiety family history	119	
Yes	44 (37.0)	
No	75 (63.0)	
Anxiety/depressive diagnoses according to DSM V		
GAD	25 (21.9)	
Panic disorder	2 (1.8)	
Agoraphobia	2 (1.9)	
Social anxiety disorder	5 (4.6)	
Medication-related anxiety	1 (0.9)	
Persistent depressive disorder	3 (2.8)	
Subsyndromal depression	15 (13.6)	

### Anxiety rates and agreement between scales

Using the established threshold of 14 and above on the PAS, 50% were classified as having anxiety (Table 2). A similar anxiety rate (54%) was found on the GAD7 with a cutoff of 4/5, indicating at least mild anxiety, with substantial agreement between the scales (Cohen’s kappa 0.62). The MDS-NMS anxiety subdomain with a cut-off of 9/10 provided anxiety rates of 55% with moderate agreement in classification with the PAS cutoff (Cohen’s kappa 0.50). On the MDS-UPDRS a cut-off of 2/3 provided a lower anxiety rate of 22% and fair agreement (Cohen’s kappa 0.36); a cut-off of 1 /2 provided an anxiety rate of 38% and also fair agreement with the PAS classification (Cohen’s kappa 0.39) but specificity was low. The single question on anxiety had low specificity and previous diagnosis of anxiety had low sensitivity, and both had only slight agreement with the PAS classification for anxiety (Cohen’s kappa 0.19 and 0.18, respectively).

**Table 2:** Anxiety rates using different methods

Variables	Frequency (%)	Agreement with PAS ≥ 14	Sensitivity*	Specificity*
Rate of self-reported anxiety “yes”	102 (85)	0.193	95%	24%
Previous diagnosis of Anxiety	24 (20)	0.175	29%	88%
Self-reported anxiety in the last week (MDS-UPDRS item 1.4 ≥ 1	98 (81.0)	0.202	92%	28%
(Self-reported anxiety in the last week (MDS-UPDRS item 1.4 ≥ 2	46 (38.0)	0.385	58%	91%
Self-reported anxiety in the last week (MDS-UPDRS item 1.4 ≥ 3	27 (22.3)	0.363	41%	95%
Self-reported anxiety in the last 4 weeks (MDS-NMS Scale Anxiety subscale severity ≥3)	106 (88.3)	0.161	97%	77%
Self-reported anxiety in the last 4 weeks (MDS-NMS Scale Anxiety subscale severity ≥10 total anxiety score)	67 (55.8)	0.501	81%	67%
Self-reported anxiety in the last 4 weeks (MDS-NMS Scale Anxiety subscale severity ≥14 total anxiety score)	46 (38.0)	0.481	63%	84%
Self-reported anxiety in the last 4 weeks (MDS-NMS Scale Anxiety subscale severity ≥22 total anxiety score)	29 (24.2)	0.361	42%	91%
Current anxiety ≥ 5 on GAD7 (2 weeks)	66 (54.1)	0.624	86%	78%
Current anxiety ≥ 10 on GAD7 (2 weeks)	24 (19.7)	0.304	36%	95%
Current anxiety ≥ 14 on PAS (4 weeks)	59 (50.0)	n.a.	n.a	n.a
Current anxiety ≥ 18 on PAS (4 weeks)	34 (27.9)	n.a.	n.a	n.a

PAS, Parkinson Anxiety Scale; GAD, Generalized Anxiety Disorder Questionnaire; MDS-UPDRS, Movement Disorder Society revision of the Unified Parkinson’s Disease Rating Scale; Values are presented as n (%). *against the PAS

### Impact on health-related quality of life

Anxiety classification using these cut-offs on the four scales was associated with worse Hr-QoL scores on both the PDQ-39 (Table 3) and, to a lesser degree, the visual analogue scale of overall health (Table 4). Previous diagnosis of anxiety was also associated with worse PDQ-39 scores but not the visual analogue scale. There was no association of reported anxiety in response to the single question with PDQ-39 or visual analogue scale scores.

**Table 3:** Health-related quality of life (Parkinson disease Questionnaire-39) scores in patients with anxiety identified on different measures

Variables	N		Parkinson’s Disease Questionnaire − 39 score			P-value	
			No		Yes		
Rate of self-reported anxiety “yes”	115		35.4 ± 27.0		38.5 ± 25.1	0.48	
Previous diagnosis of Anxiety	115		34.9 ± 23.2		49.8 ± 30.5	0.02	
Self-reported anxiety in the last week (MDS-UPDRS item 1.4 ≥ 1	116		29.4 ± 40.3		23.8 ± 25.3	0.05	
Self-reported anxiety in the last week (MDS-UPDRS item 1.4 ≥ 2	116		31.5 ± 22.2		49.1 ± 26.4	< 0.001	
Self-reported anxiety in the last week (MDS-UPDRS item 1.4 ≥ 3	116		32.7 ± 22.1		57.3 ± 26.8	< 0.001	
Self-reported anxiety in the last 4 weeks (MDS-NMS Scale Anxiety subscale severity ≥10 any anxiety item)	114		28.7 ± 20.3		46.2 ± 26.2	< 0.001	
Current anxiety ≥ 5 on GAD7 (2 weeks)	117		27.0 ± 17.7		47.3 ± 27.1	< 0.001	
Current anxiety ≥ 10 on GAD7 (2 weeks)	117		32.5 ± 21.9		60.6 ± 26.1	< 0.001	
Current anxiety ≥ 14 on PAS (4 weeks)	116		25.37 ± 17.2		51.5 ± 25.7	< 0.001	

MDS-UPDRS, Movement Disorder Society revision of the Unified Parkinson’s Disease Rating Scale; PAS, Parkinson Anxiety Scale; GAD, Generalized Anxiety Disorder Questionnaire; MDS-NMS, Movement Disorder Society Non-Motor Symptoms; Values are presented as (%)

**Table 4:** EQ-5D-5 L visual analogue scores of health in patients with anxiety identified on different measures

Variables		N	EQ-5D-5 L VAS (0 to 100)		P-value		
			No	Yes			
Rate of self-reported anxiety “yes”		116	68.9 ± 18.0	64.9 ± 17.9	0.33		
Previous diagnosis of Anxiety		116	65.8 ± 18.4	61.8 ± 15.6	0.16		
Self-reported anxiety in the last week (MDS-UPDRS item 1.4 ≥ 1		110	71.2 ± 20.0	64.0 ± 17.0	0.04		
Self-reported anxiety in the last week (MDS-UPDRS item 1.4 ≥ 2		110	68.0 ± 16.6	61.0 ± 18.8	0.05		
Self-reported anxiety in the last week (MDS-UPDRS item 1.4 ≥ 3		110	67.6 ± 17.0	57.7 ± 18.2	0.01		
Self-reported anxiety in the last 4 weeks (MDS-NMS Scale Anxiety subscale severity ≥10 any anxiety item)		115	69.3 ± 18.2	62.0 ± 16.	0.02		
Current anxiety ≥ 5 on GAD7 (2 weeks)		118	68.5 ± 17.6	62.3 ± 17.5	0.006		
Current anxiety ≥ 10 on GAD7 (2 weeks)		118	67.4 ± 17.1	56.3 ± 17.7	0.007		
Current anxiety ≥ 14 on PAS (4 weeks)		117	69.53 ± 16.7	60.6 ± 17.8	0.008		

MDS-UPDRS, Movement Disorder Society revision of the Unified Parkinson’s Disease Rating Scale; PAS, Parkinson Anxiety Scale; GAD, Generalized Anxiety Disorder Questionnaire; MDS-NMS, Movement Disorder Society Non-Motor Symptoms; Values are presented as mean ± standard deviation

## Discussion

Our results suggest that, as expected, a single question on anxiety is not a reliable or meaningful assessment method for anxiety in PD. Agreement with established, more detailed assessments is low and, whilst common, a positive answer to this question is not associated with a difference in health-related quality of life. Those with an existing diagnosis of anxiety are more likely to have impaired health-related quality of life, but sensitivity is low and others are missed. On the other hand, whilst there is remaining heterogeneity in classification between established scales to assess anxiety in PD, there is moderate to substantial agreement between established scales, using specific cut-off that have been previously published and were confirmed in this population. This includes the GAD7 and MDS-Nonmotor symptoms scale, for which we established the cutoffs corresponding to the Parkinson’s specific PAS cutoff, which we confirmed using ROC analysis. For the PAS, the Youden index confirmed an optimal cut-off value of 14, which is consistent with its validation study (Leentjens et al. 2014). The use of these cut-off in studies in PD is encouraged as they indicate a comparable level of anxiety severity on these scales. The cutoff of the GAD7 correspond to its established cutoffs for mild anxiety. There had been no previous study to identify the optimal cut-off for anxiety on the relatively new MDS-NMS scale, and we here identified 13/14 as the cut-off most comparable to the PAS cut-off, with moderate agreement with the PAS cutoff. Both of these classification were clinically meaningful as indicated by their association with impaired health-related quality of life scores. On the other hand, for the MDS-UPDRS question on anxiety we did not identify a clear corresponding cutoff score. This item is a screening question on a comprehensive larger scale, and does incorporate persistence and the degree of functional impact on daily life. As a question included in the standard assessment tool for patients with PD, it provides an indication of clinically significant anxiety, that is also associated with impairment health-related quality of life at a cut-off of 2/3. However, this under-recognises the presence of anxiety symptoms that are identified on other scales. Therefore, a lower cutoff of 1 /2, which signifies presence of persistent anxiety symptoms without interference with daily activities, can provide a screening method for anxiety symptoms better. However, for identification of anxiety in clinical studies assessment (when full psychiatric interview is precluded) the PAS, GAD7 or MDS-NMS at the established cutoffs should preferentially be used to identify those with anxiety. In summary, whilst the gold standard for assessment of anxiety in PD remains the clinical diagnosis, e.g. using DSM V criteria, the cutoff given for the assessment tools assessed in this study the PAS at the established cutoff, can serve to identify similar results and categorisation of patients with PD.
